# Regulation of immune responses in primary biliary cholangitis: a transcriptomic analysis of peripheral immune cells

**DOI:** 10.1097/HC9.0000000000000110

**Published:** 2023-04-04

**Authors:** Victoria Mulcahy, Evaggelia Liaskou, Jose-Ezequiel Martin, Prasanti Kotagiri, Jonathan Badrock, Rebecca L. Jones, Simon M Rushbrook, Stephen D. Ryder, Douglas Thorburn, Simon D. Taylor-Robinson, Graeme Clark, Heather J. Cordell, Richard N. Sandford, David E. Jones, Gideon M. Hirschfield, George F. Mells

**Affiliations:** 1 Academic Department of Medical Genetics, University of Cambridge, Cambridge, UK; 2 Cambridge Liver Unit, Cambridge University Hospitals NHS Foundation Trust, Cambridge, UK; 3 Centre for Liver and Gastrointestinal Research, National Institute for Health Research (NIHR) Birmingham Biomedical Research Centre (BRC), University Hospitals Birmingham NHS Foundation Trust and University of Birmingham, UK; 4 Institute of Immunology & Immunotherapy, University of Birmingham, Birmingham, UK; 5 Cancer Molecular Diagnostic Laboratory, Oncology Department, University of Cambridge, Cambridge, UK; 6 Cambridge Institute of Therapeutic Immunology and Infectious Diseases, Jeffrey Cheah Biomedical Centre, University of Cambridge, Cambridge, UK; 7 Department of Medicine, University of Cambridge, Cambridge, UK; 8 Leeds Liver Unit, The Leeds Teaching Hospitals NHS Trust, Leeds, UK; 9 Department of Hepatology, Norwich Medical School, University of East Anglia, Norwich, UK; 10 NIHR Nottingham BRC, Nottingham University Hospitals NHS Trust, University of Nottingham, Nottingham, UK; 11 The Sheila Sherlock Liver Centre, Royal Free London NHS Foundation Trust, London, UK; 12 Department of Surgery and Cancer, Imperial College London, St Mary’s Campus, London, UK; 13 Stratified Medicine Core Laboratory (SMCL) Next Generation Sequencing Hub, NIHR Cambridge BRC, Cambridge, UK; 14 Population Health Sciences Institute, Faculty of Medical Sciences, Newcastle University, Newcastle-upon-Tyne, UK; 15 Institute of Cellular Medicine, Newcastle University, Newcastle-upon-Tyne, UK; 16 NIHR Newcastle BRC, Newcastle University, Newcastle-upon-Tyne, UK; 17 Toronto Centre for Liver Disease, University Health Network and Department of Medicine, University of Toronto, Toronto, Canada

## Abstract

In patients with primary biliary cholangitis (PBC), the serum liver biochemistry measured during treatment with ursodeoxycholic acid—the UDCA response—accurately predicts long-term outcome. Molecular characterization of patients stratified by UDCA response can improve biological understanding of the high-risk disease, thereby helping to identify alternative approaches to disease-modifying therapy. In this study, we sought to characterize the immunobiology of the UDCA response using transcriptional profiling of peripheral blood mononuclear cell subsets.

**Methods:**

We performed bulk RNA-sequencing of monocytes and T_H_1, T_H_17, T_REG_, and B cells isolated from the peripheral blood of 15 PBC patients with adequate UDCA response (“responders”), 16 PBC patients with inadequate UDCA response (“nonresponders”), and 15 matched controls. We used the Weighted Gene Co-expression Network Analysis to identify networks of co-expressed genes (“modules”) associated with response status and the most highly connected genes (“hub genes”) within them. Finally, we performed a Multi-Omics Factor Analysis of the Weighted Gene Co-expression Network Analysis modules to identify the principal axes of biological variation (“latent factors”) across all peripheral blood mononuclear cell subsets.

**Results:**

Using the Weighted Gene Co-expression Network Analysis, we identified modules associated with response and/or disease status (*q*<0.05) in each peripheral blood mononuclear cell subset. Hub genes and functional annotations suggested that monocytes are proinflammatory in nonresponders, but antiinflammatory in responders; T_H_1 and T_H_17 cells are activated in all PBC cases but better regulated in responders; and T_REG_ cells are activated—but also kept in check—in responders. Using the Multi-Omics Factor Analysis, we found that antiinflammatory activity in monocytes, regulation of T_H_1 cells, and activation of T_REG_ cells are interrelated and more prominent in responders.

**Conclusions:**

We provide evidence that adaptive immune responses are better regulated in patients with PBC with adequate UDCA response.

## INTRODUCTION

Primary biliary cholangitis (PBC) is an autoimmune liver disease characterized by progressive destruction of the small, intra-hepatic bile ducts, leading in many cases to cirrhosis.^1^ First-line treatment of PBC is with ursodeoxycholic acid (UDCA), a hydrophilic bile acid that acts mainly by displacing hydrophobic bile acids in bile and by enhancing choleresis. In patients with PBC, the serum liver biochemistry during treatment with UDCA—the UDCA response—accurately predicts long-term outcome.^2^ Thus, patients with adequate UDCA response are at a lower risk of disease progression, whereas those with inadequate UDCA response are at a higher risk. The latter are prioritized for second-line treatment with agents that also act mainly through choleresis: the farnesoid X receptor agonist, obeticholic acid, or the peroxisome proliferator-activated receptor agonists, bezafibrate or fenofibrate.^3^ Up to 50% of patients with inadequate response to UDCA also have an inadequate response to second-line treatment, however, and so remain at risk of disease progression.^4^ Novel therapeutic approaches—not just targeting the composition or secretion of bile—are needed for such patients. Molecular characterization of patients stratified by the UDCA response can improve biological understanding of the high-risk disease, thereby helping to identify alternative approaches to disease-modifying therapy.

We have reported gene expression analysis of liver tissue from patients with high versus low-risk PBC, which showed greater biliary epithelial cell (BEC) senescence and T-cell activation in those with high-risk disease.^5^ More recently, we reported serum proteomic profiling of 526 patients with PBC stratified by UDCA response, which showed reproducible elevation of proteins with well-defined roles in inflammation and immunity, such as CCL20, CXCL11, IL-4RA, and IL-18R1, in nonresponders.^6^ These findings highlight the potential value of profiling the immune systems of patients stratified by UDCA response. A starting point is to profile the immune cell types that are thought to be dysregulated in PBC itself.

Immune cell types implicated in the pathogenesis of PBC include monocytes; T_H_1, T_H_17, and T_REG_ cells; CD8^+^ T cells, and B cells. For example, in PBC, monocytes are more sensitive to toll-like receptor signalling^7^; portal infiltrates consist mainly of CD4^+^ T cells, heavily skewed towards T_H_1 and T_H_17 cells^8^,^9^; there is insufficiency of T_REG_ cells^10^; CD8^+^ T cells infiltrate the biliary epithelia and cause segmental apoptotic destruction of cholangiocytes^11^,^12^; and B cells contribute to disease through cross-presentation of antigen and production of autoantibodies.^13^,^14^

In the current study, guided by these observations, we used bulk RNA-sequencing (RNA-seq) to profile the transcriptomes of monocytes and T_H_1, T_H_17, T_REG_, and B cells from the peripheral blood of PBC patients with an adequate response to UDCA, those with inadequate response, and healthy controls. We designed the study for case-control and within-case analyses to gain insight into the immunobiology of PBC itself and that of refractory disease. For each cell type, we sought to identify networks of co-expressed genes associated with 1 or more traits. To do this, we used an approach called weighted gene co-expression network analysis (WGCNA).^15^ The premise of this approach (and others like it) is that genes do not function in isolation but in networks^16^; genes within those networks are co-expressed; and statistical methods can be used to identify those co-expressed genes.^17^ Association can then be determined between gene co-expression networks and traits of interest; and functional annotation of associated networks can be employed to gain biological insight into those traits.

## METHODS

### Participants

Cases were adults (≥18 y of age) with an established diagnosis of PBC,^3^ who had received≥12 months of ongoing treatment with UDCA. Response to UDCA was defined by alkaline phosphatase<1.67 times the upper limit of normal.^18^ Controls were age and sex-matched healthy volunteers. For all participants, exclusion criteria were other forms of liver disease (eg, viral hepatitis), decompensated cirrhosis, HCC, liver transplantation, immunosuppression, poorly controlled diabetes mellitus, pregnancy, and alcohol misuse. Decompensated cirrhosis was defined by a total bilirubin>50 μmol/L or any occurrence of variceal hemorrhage, ascites, or HE.^19^ Cases were recruited from 6 liver treatment centers across the UK as part of the UK-PBC Nested Cohort Study.^6^ Adherence to treatment with UDCA was verified in all cases. Controls were recruited from the Cambridge NIHR BioResource (https://www.cambridgebioresource.group.cam.ac.uk/). Written informed consent was obtained from each participant. The study protocol conformed to the ethical guidelines of the 1975 Declaration of Helsinki. Ethical approval for the study was from the North-West Preston Research Ethics Committee (REC reference: 14/NW/1146).

### Samples

Up to 100 mL of blood was collected from each participant for the isolation of peripheral blood mononuclear cells (PBMCs). The isolation of PBMCs was undertaken on-site within 2 hours of phlebotomy according to a standard operating procedure. The PBMCs were stored overnight at −80°C and transferred on dry ice the next day to the University of Birmingham for long-term storage over liquid nitrogen. Please see Supplementary Data (Fig. S1, http://links.lww.com/HC9/A236) for further details.

### Sorting of PBMC subsets

Peripheral blood mononuclear cells were sorted into subsets at the University of Birmingham. The PBMCs were first thawed as described in the Supplementary Data, http://links.lww.com/HC9/A236. CD14^+^ monocytes were then selected using human CD14 MicroBeads (Miltenyi Biotec, Woking, UK) according to the manufacturer’s instructions; CD14^+^ cell pellets were frozen at −80^°^C until RNA extraction. Non-CD14^+^ cells were resuspended in PBS and stained with live/dead marker (Zombie APC-Cy7 at 1:1000; Biolegend, San Diego, CA, USA) for 20 minutes at room temperature. Cells were washed and stained with the following fluorochrome-conjugated antibodies (BD Biosciences, UK): CD3-APC, CD4-PECF594, CD127-PECy7, CD25-BB515, CXCR3-PerCP-Cy5.5, CCR6-BV421, and CD19-PE for fluorescent-associated cell sorting (using BD Aria Fusion, BD, UK) of the immune cell subsets: (1) CD19^+^CD3^–^ B cells, (2) CD3^+^CD4^+^CD25^high^CD127^low^ T_REG_ cells, (3) CD3^+^CD4^+^CCR6^+^CXCR3^–^ T_H_17 cells, and (4) CD3^+^CD4^+^CCR6^–^CXCR3^+^ T_H_1 cells (Fig. S2, http://links.lww.com/HC9/A236). We used an anti-CD3 monoclonal antibody that did not induce T-cell activation (Fig. S3, http://links.lww.com/HC9/A236). All immune cell subsets were sorted into tubes containing RPMI−1640+10% FCS media kept at 4°C during the sorting period. After sorting, cells were pelleted and resuspended in RLT+βME buffer for lysis and RNA extraction.

### RNA extraction

RNA was extracted from PBMC subsets at the University of Birmingham using the RNEasy Plus Micro and RNeasy Plus Mini Kits (Qiagen Ltd, Manchester, UK) for<500,000 and>500,000 sorted cells, respectively. As recommended, eluted RNA samples were stored at −80^°^C until RNA-seq.

### RNA-sequencing

RNA-seq was completed in the Stratified Medicine Core Laboratory at the University of Cambridge. Following quality control (QC) checks, the SMARTer Stranded Total RNA-seq Pico kit from Clontech (Mountain View, CA, USA) was used to generate cDNA libraries within 30 days of RNA extraction, and the Illumina (San Diego, CA, USA) HiSeq. 2500 and HiSeq. 4000 platforms were used to sequence them.

### Statistical analysis

We employed a standardized pipeline for QC and processing of reads. Read quality was assessed using FastQC (https://www.bioinformatics.babraham.ac.uk/projects/fastqc); alignment was performed using STAR (Spliced Transcripts Alignment to a Reference)^20^; and count matrices were generated using FeatureCounts in Rsubreads^21^ and stored as a DGEList object in edgeR^22^ for downstream analysis. Additional QC checks (including tests for batch effect) were done using DESeq2^23^ and limma (Linear models for microarray data).^24^

For each PBMC subset, we used WGCNA to identify networks of co-expressed genes associated with disease or UDCA response status. We first identified modules, which are clusters of highly interconnected genes, each representing a gene co-expression network. By convention, each module is arbitrarily allocated the name of a color. For each module, we calculated the module eigengene (ME), module membership, and gene significance. The ME is the first principal component of the module, representative of gene expression profiles in that module; module membership is the correlation coefficient for each gene with the ME; and gene significance is the correlation coefficient for each gene with the trait of interest. Finally, we tested the association of each module with each trait (ie, all cases vs. controls, nonresponders vs. controls, responders vs. controls, and responders vs. nonresponders) by calculating the eigengene significance, based on correlation between the ME and trait of interest. We defined a significant association between a module and trait as *q*<0.05, where *q* is the false discovery rate adjusted *p*-value.

For each module associated with 1 or more traits, we identified hub genes and looked for enrichment of functional annotations. Hub genes are the genes with the highest connectivity within the module (ie, the genes most strongly co-expressed with the greatest number of other genes). In general, we identified hub genes as being among the 30 most connected genes in the module, as well as having module membership≥3rd quartile and gene significance≥3rd quartile. We used the Enrichr platform^25^ to look for the enrichment of functional annotations. For brevity and consistency, only Hallmark gene sets^26^ are reported in this manuscript. For each pairwise comparison, we also performed Gene Set Enrichment Analysis (GSEA)^27^ of normalized count data from each PBMC subset. As recommended at the GSEA platform (https://www.gsea-msigdb.org/gsea/index.jsp), we took *q*<0.25 to define significant enrichment.

Finally, recognizing that each PBMC subset is part of a unified immune system, we used Multi-Omics Factor Analysis (MOFA)^28^ for integrated analysis of all modules identified across all PBMC subsets. By this approach, we decomposed the combined data into a small number of latent factors (LFs) and used ANOVA to identify the LFs associated with trait status (ie, control, responder, or nonresponder). To characterize the LFs associated with trait status, we identified the modules with the heaviest loading on each of these factors. Please see the Supplementary Data, http://links.lww.com/HC9/A236, for further details.

## RESULTS

### Participants and samples

We analyzed samples from 15 responders (median alkaline phosphatase 0.87×ULN, interquartile range [IQR] 0.75–1.00); 16 nonresponders (median alkaline phosphatase 3.68×ULN, IQR 3.14–4.48); and 15 disease-free controls. These groups were well-matched except for PBC status or activity (Table 1, Fig. S4, http://links.lww.com/HC9/A236). Vibration-controlled transient elastography measurements were comparable in responders and nonresponders. Only 1 participant, a responder, had cirrhosis according to the Vibration-controlled transient elastography thresholds proposed by Corpechot et al (2012).^29^ Cell counts are shown in Fig. S5, http://links.lww.com/HC9/A236. The median RNA integrity number (RIN) of the samples was 8.9 (IQR 8.1–9.2). Following QC checks, the mean number of raw reads per sample was 145 million; the number of mapped reads was 43 million; the percentage PCR duplicates was 37%; and the number of genes at 1 fragment per kilobase of transcript per million mapped reads, was 14,000. MA plots showed no evidence of systematic bias in the RNA-seq output (Fig. S6, http://links.lww.com/HC9/A236). We confirmed the identity of each cell subset by high expression of characteristic genes (Fig. S7, http://links.lww.com/HC9/A236). Multidimensional scaling of count data confirmed similarity within PBMC subsets and dissimilarity between them (Fig. S8, http://links.lww.com/HC9/A236).

**TABLE 1:** Participant characteristics

	Nonresponders (n=16)	Responders (n=15)	Controls (n=15)	*p*-value
Age (y)	60 (56–66)	65 (54–71)	57 (31–74)	0.2
No. of women (%)	14 (87.5%)	15 (94%)	15 (100%)	0.4
Disease duration (y)	10.8 (6.7–13.8)	8.3 (3.2–12.5)	—	0.3
UDCA dose (mg/kg/day)	14.3 (13.2–16.0)	11.9 (10.3–13.8)	—	7.2×10^−3^
ALP (U/L)	467.5 (383.8–541.5)	97.0 (83.5–120.0)	64 (53–78)	2.3×10^−6^
ALP/ULN	3.7 (3.1–4.5)	0.8 (0.7–1.0)	0.5 (0.4–0.6)	2.3×10^−6^
ALT (U/L)	60.5 (47–81.5)	31 (19.5–49.5)	19 (16–28)	5.3×10^−3^
Bilirubin (μmol/L)	12 (9.8–19.3)	7 (6–10)	10 (9–11)	1.4×10^−3^
Albumin (g/L)	38 (36–40.3)	45.0 (42.5–47.5)	39 (37–40)	4.5×10^−4^
Platelets (10^9^/L)	250 (203.5–303.5)	245 (190–322.5)	224 (198–300)	0.9
Transient elastography (kPa)	6.8 (5.6–8.0)	7.6 (5.0–13.9)	—	0.7

*Notes:* Median values (interquartile range) are shown for all parameters except for the number (percentage) of women.

Statistical tests were ANOVA for comparison of age across all 3 groups; the Kruskal-Wallis chi-square test to compare the proportion of women in each group; and a 2-tailed Mann-Whitney test to compare all other parameters in nonresponders versus responders.

Abbreviations: ALP, alkaline phosphatase; UDCA, Ursodeoxycholic acid.

**FIGURE 1:**
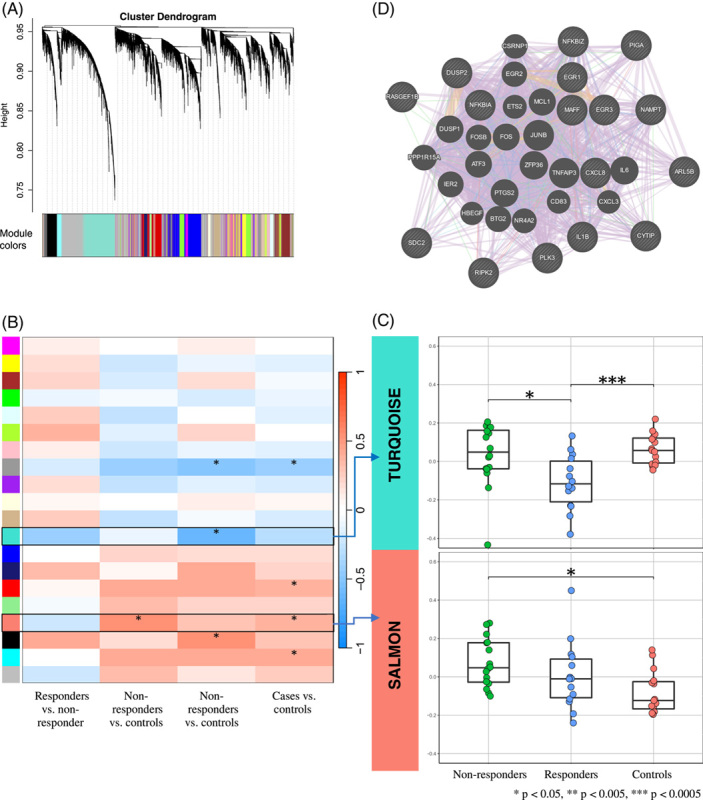
Weighted gene co-expression network analysis (WGCNA) of RNA-sequencing data from CD14 cells. (A) Dendrogram showing gene co-expression networks (‘modules’). (B) Heatmap showing the strength of correlation between each module (rows) and each trait (columns). Asterisks indicate that the module is associated with the trait at *q*<0.05, where *q* is the false discovery rate-adjusted *p*-value. Rows outlined in black identify modules with at least moderate correlation (r≥0.5) with 1 or more traits and enriched with Hallmark gene sets at *q*<0.05. (C) Boxplots of the module eigengenes (ie, the first principal component of gene expression in the module; y-axis) in each group of participants (x-axis). Asterisks indicate the statistical difference between the module eigengene significance (based on the correlation between the module eigengene and trait of interest) and the trait comparison groups. Asterisks indicate the corresponding *p* values. (D) GeneMANIA protein-protein interaction plot showing protein-coding–hub genes in the salmon module for nonresponders versus controls. Protein-coding–hub genes are shown with cross-hatched circles of a uniform size, while those that were added as relevant genes by GeneMANIA are shown with solid circles whose size is proportional to the number of interactions they have. Lines correspond to the type of interactions. Purple: Genes known to be co-expressed in existing gene databases. Pink: Proteins known to be linked. Turquoise: Genes present in a shared annotated pathway. Blue: Genes expressed in the same tissue. Orange: Predicted functional relationships between genes. Green: Predicated genetic interactions. Olive: Shared protein domains. Abbreviations: WGCNA, Weighted gene co-expression network analysis.

Using WGCNA, we identified 19 modules (ie, networks of co-expressed genes) in monocytes (Figure 1A). Of these, 6 were correlated at *q*<0.05 with 1 or more traits (ie, case vs. control, nonresponder vs. control, responder vs. control, or nonresponder vs. responder) (Figure 1B). Among the other PBMC subsets, 7 of 11 modules in T_H_1 cells; 6 of 11 modules in T_H_17 cells; 5 of 9 modules in T_REG_ cells; and 5 of 10 modules in B cells were also correlated at *q*<0.05 with 1 or more traits. Table 2 lists modules showing at least moderate correlation (r≥0.5) with 1 trait or another.

**TABLE 2:**
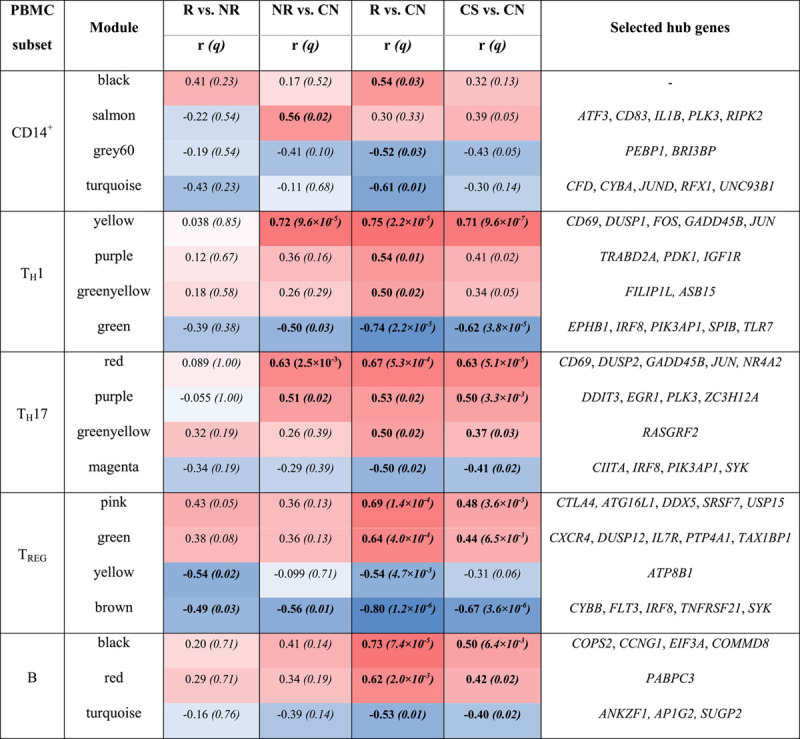
Modules of co-expressed genes

*Notes:* Modules are shown that were at least moderately correlated (r≥0.5) with one or more traits.

Bold indicates correlation at q<0.05.

Selected hub genes are those which readily describe the module.

Abbreviations: NR, non-responder; PBMC, peripheral blood mononuclear cell; R, responder.

In monocytes, the salmon module was positively correlated with nonresponders versus controls (r=0.56, *q*=0.023). This module was strongly enriched for TNF signalling (Table 3). Hub genes (ie, the most highly connected genes in the module) included *CD83*, an activation marker for Ag-presenting cells; *RIPK2*, a potent activator of NF-кB; and *IL1B*, a key mediator of inflammation (Table 2, Figure 1D). In contrast, the turquoise module was negatively correlated with responders versus controls (r=−0.61, *q*=7.7×10^−3^). Hub genes in this module included *JUND*, an activator protein (AP)-1 transcription factor; and *CYBA*, which is essential for the generation of superoxide in phagocytes. As shown in Figure 1C, genes in the salmon module were upregulated in nonresponders compared with controls (*p* = 1.2×10^−3^), whereas those in the turquoise module were downregulated in responders compared with controls (*p* = 3.8×10^−4^). This suggests a predominance of activated and proinflammatory monocytes in nonresponders, in contrast to a predominance of inactivated monocytes in responders.

**TABLE 3:** Pathway enrichment

				Enrichr		
PBMC subset	Module	Comparison group direction of effect in WGCNA		Hallmark pathway	*p*	q
CD14^+^	Salmon	—	—	TNFα signalling through NF-кB	1.75×10^−52^	7.89×10^−51^
		—	—	Apoptosis	3.45×10^−6^	3.58×10^−5^
		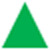 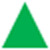	NR vs. CN	mTORC1 signalling	4.78×10^−6^	3.58×10^−5^
		—	—	KRAS signalling up	4.78×10^−6^	3.58×10^−5^
		—	—	IL-2/STAT5 signalling	2.56×10^−6^	1.51×10^−4^
		—	—	IFNγ response	2.69×10^−6^	1.51×10^−4^
T_H_1	Yellow	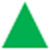 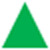 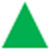	NR vs. CN	TNFα signalling via NF-кB	2.35×10^−39^	1.08×10^−37^
		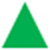 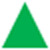 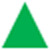	R vs. CN	p53 pathway	2.36×10^−4^	3.63×10^−3^
		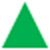 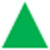 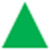	CS vs. CN	Apoptosis	5.30×10^−4^	6.10×10^−3^
	Green	—	—	—	—	—
		—	IL-2/STAT5 signalling	2.32×10^−4^	2.77×10^−3^	—
		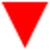 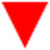 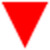	R vs. CN	IFNγ response	2.46×10^−4^	2.77×10^−3^
		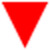 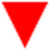	NR vs. CN	IL-6/JAK/STAT3 signalling	1.83×10^−3^	9.94×10^−3^
		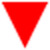 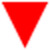	CS vs. CN	TNFα signalling through NF-кB	1.99×10^−3^	9.94×10^−3^
		—	—	KRAS signalling up	1.99×10^−3^	9.94×10^−3^
T_H_17	Purple	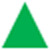 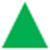	NR vs. CN	TNFα signalling through NF-кB	3.35×10^−6^	8.71×10^−5^
		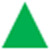 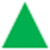	R vs. CN	—	—	—
		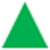 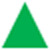	CS vs. CN	—	—	—
	Red	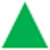 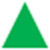	NR vs. CN	TNFα signalling via NF-кB	2.04×10^−30^	9.40×10^−29^
		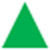 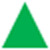	CS vs. CN	IFNγ response	3.72×10^−6^	4.27×10^−5^
		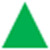 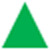	R vs. CN	IL-2/STAT5 signalling	1.52×10^−5^	1.40×10^−4^
	Magenta	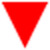 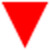	R vs. CN	KRAS signalling up	1.13×10^−9^	2.08×10^−8^
		—	—	IFNγ response	6.48×10^−5^	4.79×10^−4^
		—	—	IL-6/JAK/STAT3 signalling	1.40×10^−4^	8.60×10^−4^
T_REG_	Green	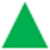 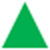	R vs. CN	TNFα signalling through NF-кB	1.74×10^−2^	6.43×10^−1^
	Brown	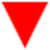 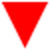	R vs. NR	KRAS signalling up	1.44×10^−6^	6.77×10^−5^
		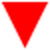 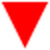	NR vs. CN	IL-6/JAK/STAT3 signalling	8.10×10^−6^	1.27×10^−4^
		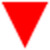 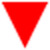 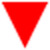	R vs. CN	IL-2/STAT5 signalling	1.33×10^−5^	1.36×10^−4^
		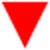 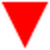	CS vs. CN	TNFα signalling through NF-кB	4.27×10^−5^	2.87×10^−4^
B	Black	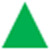 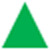 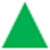	R vs. CN	TNFα signalling through NF-кB	2.60×10^−4^	1.09×10^−2^
		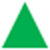 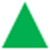	CS vs. CN	—	—	—

*Notes:* Enrichment of Hallmark pathways was evaluated using the platform, Enrichr.

Pathways enriched at *q*<0.05 are shown.

The direction of effect for the comparison groups is highlighted.

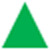
indicates positive correlation,
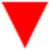
indicates negative correlation.

q is the FDR corrected *p*-value.

▴▴indicates moderate correlation ▴▴▴indicates strong correlation.

Abbreviation: FDR, false discovery rate; NR, non-responder.

In T_H_1 cells, the yellow module showed a strong positive correlation with both responders (r=0.75, *q*=2.2×10^−5^) and nonresponders (r=0.72, *q*=9.6×10^−5^) versus controls (Table 2). This module was enriched for TNF signalling and contained hub genes such as *CD69*, an activation marker; *JUN* and *FOS*, which form the mitogenic AP-1 transcription factor; and *GADD45B*, which activates MAP kinases. Conversely, the green module showed a strong negative correlation with responders versus controls (r=−0.74, *q*=2.2×10^−5^) and a moderate negative correlation with nonresponders versus controls (r=−0.5, *q*=0.03). This module was enriched for IL-2, IFNγ, and IL-6 signalling. Hub genes included *IRF8*, a transcription factor that promotes T_H_1 cells.^30^ Genes in the yellow module were upregulated in both responders and nonresponders, whereas those in the green module were downregulated in responders more than in nonresponders (Fig. S10, http://links.lww.com/HC9/A236). This suggests that T_H_1 cells are activated in all patients with PBC (irrespective of UDCA response), with stronger regulation of these cells in responders.

In T_H_17 cells, the red module was correlated with responders (r=0.67, *q*=5.30×10^−4^) as well as nonresponders (r=0.63, *q*=2.46×10^−3^) versus controls (Table 2). It was enriched for TNF, IFNγ, and IL-2 signalling; hub genes included *JUN*, *GADD45B*, and *NR4A2*. The purple module was also correlated with both responders (r=0.53, *q*=0.015) and nonresponders (r=0.51, *q*=0.024) versus controls. Hub genes in this module included *EGR1*, which induces transcription of T-bet^31^ and *ZC3H12A*, which is critical for IL-17-mediated inflammation.^32^ In contrast, the magenta module was negatively correlated with responders versus controls (r=−0.50, *q*=0.015). It was enriched for KRAS, IL-6, and IFNγ signalling; and contained the hub gene, *PIK3AP1*, which is required for the generation of pathogenic T_H_17 cells.^33^ Genes in the red and purple modules were upregulated in responders and nonresponders, whereas those in the magenta module were downregulated in responders (Fig. S11, http://links.lww.com/HC9/A236). These findings suggest that T_H_17 cells are activated in all PBC patients—but may be better regulated in responders.

In T_REG_ cells, the pink module was correlated with responders versus controls (r=0.69, *q*=1.4×10^−4^). Hub genes in this module notably included the immune checkpoint, *CTLA4*. The green module was also correlated with responders versus controls (r=0.64*, q*=4.0 ×10^−4^). It was enriched for TNF signalling and contained the hub genes, *CXCR4* and *IL7R*. Conversely, the brown module showed a strong negative correlation with responders versus controls (r=−0.80, *q*=1.17×10^−6^); moderate negative correlation with nonresponders versus controls (r=−0.56, *q*=0.011); and a moderate negative correlation with responders versus nonresponders (r=−0.49, *q*=0.026) (Fig. S12, http://links.lww.com/HC9/A236). This module was enriched for KRAS, IL-6, IL-2 signalling, and TNF signalling; and contained the hub gene, *IRF8*, the identity-keeper for suppressive T_H_1-like T_REG_ cells^34^ (Fig. S12H, http://links.lww.com/HC9/A236). Genes in the pink and green modules were upregulated in responders versus controls, whereas those in the brown module were downregulated in responders and nonresponders versus controls and responders versus nonresponders. These observations suggest that T_REG_ cells are more active—but also kept in check—in responders.

In B cells, the black module showed a strong correlation with responders versus controls (r=0.73, *q*=7.37×10^−5^). This module was enriched with genes involved in TNF signalling.

### Gene set enrichment analysis

We found that GSEA was broadly consistent with WGCNA. In monocytes, for example, GSEA showed positive enrichment of proinflammatory pathways in both responders and nonresponders versus controls—but also showed positive enrichment of MYC signalling and oxidative phosphorylation in responders versus nonresponders. In T_H_1 cells, GSEA showed positive enrichment of TNF signalling in nonresponders versus controls (implying activation)—but negative enrichment of IL-2 and IL-6 signalling in responders and nonresponders versus controls, and negative enrichment of IFNγ signalling in responders versus controls (implying regulation) (Fig. S15, http://links.lww.com/HC9/A236). In T_REG_ cells, GSEA showed positive enrichment of MYC signalling (which encourages the proliferation of T_REG_ cells) in responders versus controls—but negative enrichment of TGFβ and IL-2 signalling in both responders and nonresponders versus controls (Fig. S17, http://links.lww.com/HC9/A236). Thus, GSEA reiterated a balance between proinflammatory and immunoregulatory processes in PBC, favoring the latter in responders. Please see Supplementary Data (Fig. S14-S18, http://links.lww.com/HC9/A236) for further details.

### Multi-omics factor analysis

**FIGURE 2:**
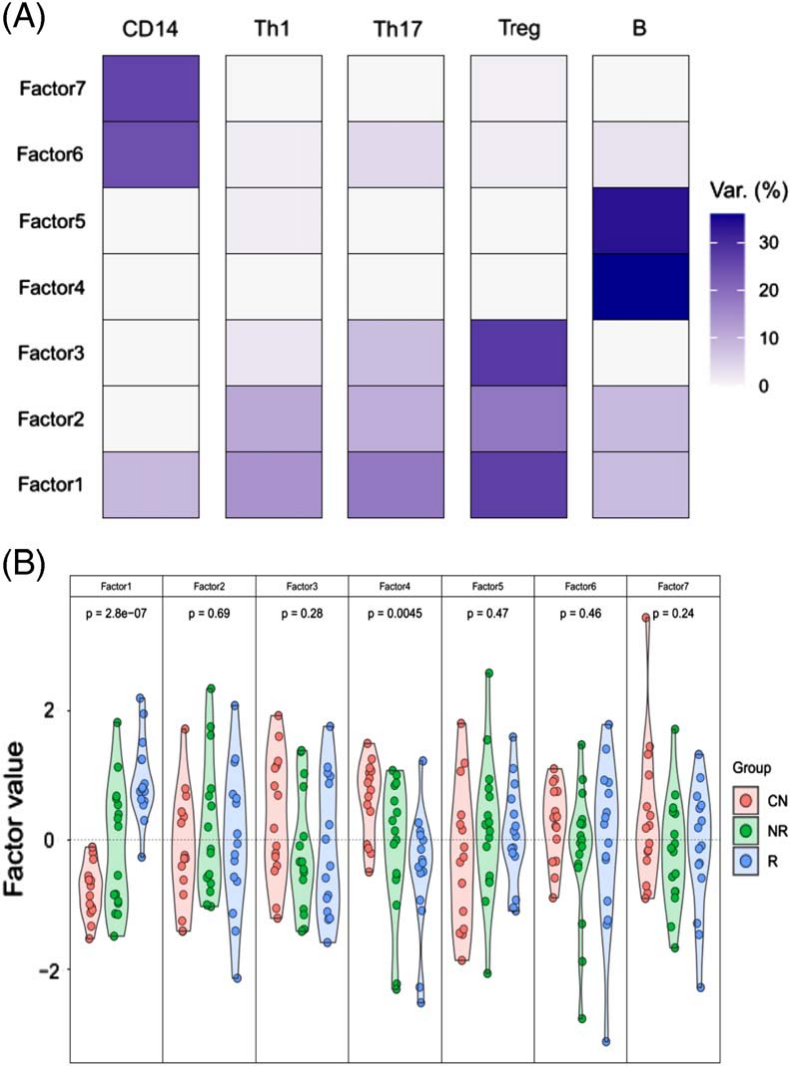
Multi-omics latent factor analysis (MOFA) of modules identified by weighted gene co-expression network analysis (WGCNA) for all cell types. (A) Heatmap to show the fitted MOFA model, displaying the percentage of variance explained for each factor (rows) in each cell type. (B) Violin plots representing the distribution of the factor values for each patient group and each latent factor (1–7). ANOVA was used to identify latent factors associated with trait status; the *p*-value for each latent factor is shown. Abbreviations: MOFA, Multi-omics latent factor analysis; WGCNA, Weighted gene co-expression network analysis.

In WGCNA and GSEA, we observed an overarching theme of immune cell activation counterbalanced by regulation. To confirm this, we used MOFA to identify the principal axes of variation across all PBMC subsets. We identified 7 latent factors accounting for most of the variance in each dataset (Figure 2A). Of these, LF1 and LF4 were associated with trait status at *p* = 2.8×10^−7^ and *p* = 0.0045, respectively. LF1 was active in all cell types. It was most active in T_REG_ cells, however, accounting for 27% of the total variance observed in this PBMC subset, compared with 18% in T_H_17 cells, 14% in T_H_1 cells, 9% in CD14^+^ cells, and 8% in B cells. LF4 was active only in B cells, accounting for 34% of the variance in this PBMC subset. The modules with the heaviest loading on LF1 or LF4 are listed in Table 4. Broadly, LF1 reaffirmed a balance between the activation and regulation of immune processes across PBMC subsets, favoring regulation. Thus, LF1 showed a strong correlation with the pink and green modules in T_REG_ cells, representing suppressive activity; a negative correlation with the turquoise module in monocytes and green module in T_H_1 cells, representing immune suppression; and only a modest correlation with the yellow module in T_H_1 cells and red module in T_H_17 cells, representing proinflammatory activity. Notably, LF1 showed a stronger association with responders, implying stronger regulation of innate and adaptive immune responses in this group of patients. The modules accounting for LF4 were enriched with genes involved in TNF signalling.

**TABLE 4:** Modules associated with LF1 and LF4

Latent factor	PBMC subset	Module	Weight	r	*p*
1	CD14^+^	Black	0.61	0.56	5.4×10^−5^
	—	Turquoise	−0.66	−0.74	8.1×10^−9^
1	T_H_1	Yellow	0.44	0.51	3.0×10^−4^
	—	Green	−0.56	−0.62	4.7×10^−6^
1	T_H_17	Red	0.45	0.55	9.7×10^−5^
	—	Greenyellow	0.63	0.69	1.4×10^−7^
1	T_REG_	Brown	−0.57	−0.61	6.2×10^−6^
	—	Green	0.66	0.74	5.8×10^−9^
	—	Pink	0.88	0.86	3.4×10^−14^
1	B	Red	0.39	0.60	1.6×10^−5^
	—	Black	0.46	0.76	2.5×10^−9^
4	B	Black	−0.65	−0.86	3.4×10^−12^
	—	Brown	−0.91	−0.91	<2.2×10^−16^
	—	Turquoise	0.93	0.94	<2.2×10^−16^

*Notes:* Modules with the greatest loading on latent factors 1 or 4 are shown.

The weight measures how strongly the module relates to the latent factor (scale from −1 to 1).

r is the correlation coefficient between the modules and the latent factor, with the associated *p*-value.

## DISCUSSION

**FIGURE 3:**
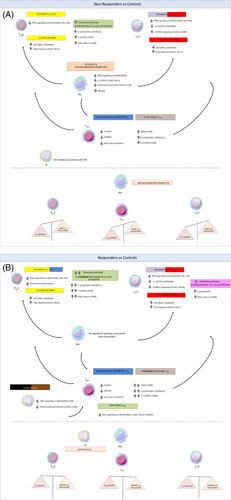
UDCA nonresponders show immunological differences compared to responders. Schematic illustrating the significant pathways correlated with nonresponders versus controls (A) and responders versus controls (B) overall highlighting the key findings of this study: (1) that monocytes are activated and proinflammatory in nonresponders; (2) T_H_1 and T_H_17 cells are activated in nonresponders and responders, but there is stronger regulation of both in the latter; (3) T_REG_ cells exhibit greater activation counterbalanced by greater regulation in responders; and (4) B cells are activated in responders.

In this study, we profiled the transcriptomes of monocytes and T_H_1, T_H_17, T_REG_, and B cells from the peripheral blood of patients with PBC with an adequate response to UDCA, those with inadequate response, and healthy controls to gain insight into the immunobiology of the UDCA response. Our data suggest that: (1) monocytes are proinflammatory in nonresponders, but antiinflammatory in responders; (2) T_H_1 and T_H_17 cells are activated in all PBC patients, but there is stronger regulation of these cell types in responders; (3) T_REG_ cells show greater activation counterbalanced by greater regulation in responders; and (4) B cells show greater activation in responders (Figure 3). Our data imply stronger regulation of immune responses in patients with a well-controlled disease.

Previous studies have shown that transcriptional profiling of PBMC subsets can provide insight into the pathogenesis of immune traits.^35^–^38^ This is the first study, however, to use transcriptional profiling of PBMC subsets to identify networks of genes associated with UDCA response or nonresponse in PBC. We employed WGCNA, a well-established method for identifying gene networks associated with traits of interest. The major advantage of WGCNA is its use of a weighted approach to identify indirect as well as direct relationships between genes, enabling robust and informative gene clustering. Strong correlations between genes are emphasized at the expense of weak correlations, which helps to identify the most highly connected (and thus most representative) genes within each cluster—so-called “hub genes”. We then used MOFA for integrated analysis of the modules identified across all PBMC subsets. MOFA can be viewed as a statistically rigorous generalization of principal component analysis to multi-omics data, which identifies the principal axes of variation across distinct data modalities in terms of latent factors. Its advantage over corresponding methods is that MOFA quantifies the proportion of variance explained by each source of variability (ie, each latent factor) in each data modality, enabling the identification of factors shared across multiple data modalities, as well as those unique to just one.

Previous studies have shown the hyper-reactivity of monocytes in PBC.^7^ Consistent with this, our data suggest that monocytes are proinflammatory in nonresponders. For example, the salmon module—enriched with proinflammatory gene sets—was correlated with nonresponders. In contrast, GSEA showed positive enrichment of MYC signalling and oxidative phosphorylation in responders compared with nonresponders. In monocytes, MYC signalling promotes M2 polarization,^39^ while oxidative phosphorylation is the primary source of energy in M2 macrophages.^40^ Thus, our findings also suggest that monocytes might be antiinflammatory in responders. One possibility, albeit speculative, is that persistent cholestasis (and the inflammatory signals associated with this) might influence the behavior of monocytes—and both, in turn, might influence adaptive immune responses.

Our data suggest that T_H_1 cells are activated in all PBC patients, irrespective of UDCA response, but they are regulated better in responders. Thus, in T_H_1 cells, the yellow module—enriched for TNF signalling, which enhances T-cell proliferation^41^—was correlated with both responders and nonresponders. Conversely, the green module—enriched for IFNγ signalling, which promotes T_H_1 cell differentiation^42^,^43^—was *negatively* correlated just with responders. We made comparable observations in T_H_17 cells: activation in all patients but stronger regulation in responders. Consistent with this, we found evidence for greater activity of T_REG_ cells in responders. For example, in T_REG_ cells, the green module containing hub genes, such as *CXCR4* and *IL7R*, was correlated with responders. Paradoxically, the brown module—enriched for IL-2 signalling, which causes expansion of T_REG_ cells—was *negatively* correlated with responders versus nonresponders, and responders versus controls. This might represent negative feedback, however, which keeps activated T_REG_ cells in check. We might expect such feedback to be more prominent in those with well-controlled disease.

Also paradoxical, our results suggest that there is greater activation of B cells in responders. Thus, the black module, enriched for TNF signalling, showed the strongest correlation with responders. B cells can promote regulatory T-cell differentiation^44^; and in a murine model of PBC, B-cell depletion exacerbates cholangitis.^45^ Therefore, it is plausible that B cells might have a protective role in PBC. This requires further exploration, especially the relationship between B and T_REG_ cells in this condition.

Collectively, our findings suggest that immune responses are better contained in responders than nonresponders. One possibility is that UDCA has direct immunomodulatory effects on the immune cells tested in this study; this seems unlikely given the breadth of effects observed. Our findings might however be consistent with the BEC senescence hypothesis. Thus, when UDCA fails to ameliorate BEC senescence, proinflammatory signals from the liver continue to drive the monocyte and T_H_1/T_H_17 cell activation seen in this study. Conversely, when UDCA-induced choleresis is effective, BEC senescence is diminished, proinflammatory signals are curtailed, and regulatory mechanisms supervene. Our findings in MOFA support this concept in that LF1 showed a stronger association with responders, favored immunoregulatory over proinflammatory activity, and was most active in T_REG_ cells. This has implications for drug development in PBC: there is clearly an important role for anti-cholestatic therapies that diminish BEC senescence—but there may also be a role for agents that target the immune processes downstream of BEC senescence.

We acknowledge the limitations of this study. The cross-sectional study design enabled us to identify associations but not causality. Even so, our study provides insight into the immunobiology of well-controlled PBC, highlighting regulatory mechanisms that could be explored for therapeutic potential. A frequent criticism of transcriptional profiling is that the genome-wide correlation between expression levels of mRNA and protein is poor. Correlation between mRNA and protein is stronger for differentially expressed genes, however, supporting the view that differential gene expression has biological meaning. We did not profile CD8^+^ T cells in the current study, even though CD8^+^ T cells are involved in the destruction of BECs in PBC.^46^ There were technical constraints on the number of PBMC subsets that could be sorted, however, so we prioritized T-helper and B-cell subsets to study immunoregulatory phenotypes. We acknowledge the importance of profiling CD8^+^ T cells (and other immune cell types, such as NK cells) in future work. Finally, we profiled circulating immune cells rather than liver infiltrates. We acknowledge that the immunopathogenic functionality and spatial array of effector and regulatory populations within inflammatory infiltrates in the liver are important to determine response to therapy in PBC. Transcriptional profiling of these populations has only recently become tractable, however, using novel approaches such as spatial transcriptomics. We look forward to using these approaches in the future.

In conclusion, this is the first study to profile the transcriptomes of PBMC subsets in patients with PBC stratified by UDCA response. Our data suggest immunological differences between responders and nonresponders—and suggest that immune cells are better restrained in PBC patients with well-controlled disease.

## Supplementary Material
