# Supplementary material for: Regulation of immune responses in primary biliary cholangitis: a transcriptomic analysis of peripheral immune cells
Source: Hepatol Commun. 2023 Apr 4;7(4):e0110. doi: 10.1097/HC9.0000000000000110 (PMC10079354; doi:10.1097/HC9.0000000000000110)

Supplementary material

**Association of preoperative interleukin-6 levels with overt hepatic encephalopathy in cirrhotic patients after TIPS**

JinYu Li 1†, Yang Liu2†, ManBiao Li2, XueTong Rong3, Zhen Yuan3, Chao Ren2, Shuai Liu1, Lei li1, Chao zhao1, Long Gao1*, DuiPing Feng1*

1Department of Oncological and Vascular Intervention, First Hospital of Shanxi Medical University, Taiyuan 030001, China.

2 College of Medical Imaging, Shanxi Medical University, Taiyuan 030001, China.

3 College of public health, Shanxi Medical University, Taiyuan 030001, China.

† These authors contribute equally to this work.

* Corresponding author.

JinYu Li, [lijinyu92@126.com](mailto:lijinyu92@126.com);

Yang Liu, [liuyang5419@163.com](mailto:liuyang5419@163.com);

ManBiao Li, [2638642822@qq.com](mailto:2638642822@qq.com):

XueTong Rong, [rrrxtxtr@163.com](mailto:rrrxtxtr@163.com);

Zhen Yuan, [yz1246391130@163.com](mailto:yz1246391130@163.com):

Chao Ren, [330209859@qq.com](mailto:330209859@qq.com);

Shuai Liu, liushuai198@163.com;

Lei li, 807967108@qq.com;

Chao zhao, zhaochaoxy@163.com;

Long Gao, Corresponding author, [gaolong@sxmu.edu.cn](mailto:gaolong@sxmu.edu.cn);

Duiping Feng, Corresponding author, [fengdp@sxmu.edu.cn](mailto:fengdp@sxmu.edu.cn);

| Table S1. Associations between serum IL-6 levels and overt hepatic encephalopathy risk by logistic regression in different etiology of cirrhosis. | | | | | | |
| --- | --- | --- | --- | --- | --- | --- |
|  | Viral | | Alcoholic | | Biliary | |
|  | RR (95%CI) | P | RR (95%CI) | P | RR (95%CI) | P |
| OHE | 5.727 (1.645-19.942) | 0.006 | 5.333 (0.468-60.797) | 0.178 | 1.111 (0.112-10.986) | 0.928 |
| OHE, overt hepatic encephalopathy. The bold presented a p-value less than 0.05. | | | | | | |

| TableS2. Receiver operating characteristic curve analysis of IL-6 and other indexes in subgroup (no history of HE). | | | | | |
| --- | --- | --- | --- | --- | --- |
| Parameter | IL-6 | Age | Child-Pugh score | CRP | Blood ammonia |
| AUC | 0.809 | 0.693 | 0.590 | 0.572 | 0.562 |
| 95%CI | 0.730-0.889 | 0.597-0.788 | 0.481-  0.698 | 0.449-0.695 | 0.403-0.721 |
| Sensitivity | 89.5％ | 73.7% | 86.8% | 42.1% | 44.4% |
| Specificity | 57.0％ | 65.8% | 34.2% | 82.3% | 74.0% |
| P value | <0.001 | 0.001 | 0.117 | 0.250 | 0.081 |
| AUC, Areas under curve. CRP, C-reactive protein. IL-6, Interleukin-6. | | | | | |

| TableS3. Pearson correlation analysis of IL-6 with Child-Pugh score  and blood ammonia | | | |
| --- | --- | --- | --- |
|  | | Blood ammonia | Child-Pugh score |
| IL-6 | r | 0.75 | 0.165 |
|  | p | 0.523 | 0.067 |

IL-6, Interleukin-6.

| TableS4.Associations between serum IL-6 levels and overt hepatic encephalopathy risk by COX regression | | |
| --- | --- | --- |
| Variables | Multivariable COX regression analysis | |
|  | HR (95%CI) | P |
| IL-6>10.5 pg/mL | 4.398 (2.158-8.960) | <0.001 |

Figure S1. The onset time of HE is following


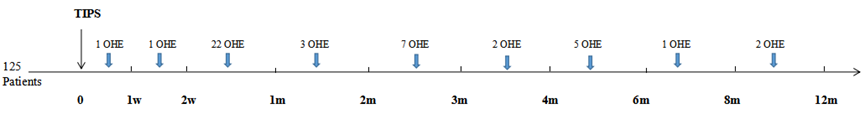

Supplement: Supplementary file 1 [file hc9-7-e0110-s001.docx]
